# Autophagy and Autophagy-Related Proteins in CNS Autoimmunity

**DOI:** 10.3389/fimmu.2017.00165

**Published:** 2017-02-27

**Authors:** Christian W. Keller, Jan D. Lünemann

**Affiliations:** ^1^Institute of Experimental Immunology, Laboratory of Neuroinflammation, University of Zürich, Zürich, Switzerland; ^2^Department of Neurology, University Hospital Zürich, Zürich, Switzerland

**Keywords:** autophagy pathways, non-canonical autophagy, multiple sclerosis, EAE/MS, oligodendrocyte death, antigen presentation

## Abstract

Autophagy comprises a heterogeneous group of cellular pathways that enables eukaryotic cells to deliver cytoplasmic constituents for lysosomal degradation, to recycle nutrients, and to survive during starvation. In addition to these primordial functions, autophagy has emerged as a key mechanism in orchestrating innate and adaptive immune responses and to shape CD4^+^ T cell immunity through delivery of peptides to major histocompatibility complex (MHC) class II-containing compartments (MIICs). Individual autophagy proteins additionally modulate expression of MHC class I molecules for CD8^+^ T cell activation. The emergence and expansion of autoreactive CD4^+^ and CD8^+^ T cells are considered to play a key role in the pathogenesis of multiple sclerosis (MS) and its animal model experimental autoimmune encephalomyelitis. Expression of the essential autophagy-related protein 5 (Atg5), which supports T lymphocyte survival and proliferation, is increased in T cells isolated from blood or brain tissues from patients with relapsing-remitting MS. Whether Atgs contribute to the activation of autoreactive T cells through autophagy-mediated antigen presentation is incompletely understood. Here, we discuss the complex functions of autophagy proteins and pathways in regulating T cell immunity and its potential role in the development and progression of MS.

## Introduction

Organisms with subcellular compartmentalization and membrane-bound organelles face constant challenges in order to maintain metabolic integrity and homeostasis. Autophagy comprises a set of evolutionary conserved catabolic pathways that converge in the guided direction of assigned cargo to the endolysosomal system (1–3). At first, autophagy was predominantly recognized for its contribution in keeping energy homeostasis, and degradation of aberrant protein aggregates (4, 5) as well as mediator of development-associated forms of cell death (6). In recent years, however, autophagy’s function has exceeded the originally allotted role as a mere protein degradation system alongside the proteasomal machinery. In addition to regulating cellular proteostasis and cell death, autophagy pathways are increasingly being recognized for actively participating in physiological and pathological immune responses. In doing so, autophagy pathways limit intracellular proliferation of pathogens (7), restrict secretion of proinflammatory mediators (8), and tweak T cell responses by orchestrating both loading and preservation of antigens on and surface-expression of antigen-presenting molecules (9–12).

Multiple sclerosis (MS) is a chronic inflammatory disease of the central nervous system (CNS), which is driven by a complex interplay of genetic, environmental, metabolic, and immunological factors. The strong genetic association between MS and the major histocompatibility complex (MHC) allele DRB1*1501 suggests that CD4^+^ T cell-mediated antigen responses play a key role in its pathogenesis. This notion is supported by the presence of clonally expanded T and B cells in MS lesions and cerebrospinal fluid samples derived from MS patients (13–15). In this review, we will first illustrate the biology of distinct autophagy pathways and their function in regulating adaptive immunity in order to discuss how the autophagy machinery potentially interferes with pathogenic adaptive immune responses in MS.

## Autophagy

Canonical autophagy implicates at least three distinct pathways: macroautophagy (MA), microautophagy (MI), and chaperone-mediated autophagy (CMA) (16). Although all of the above mentioned pathways coalesce in the lysosome, they considerably differ (albeit showing some overlap), in their means of cargo transportation, triggering events, and regulatory factors.

## Macroautophagy

**Figure 1:**
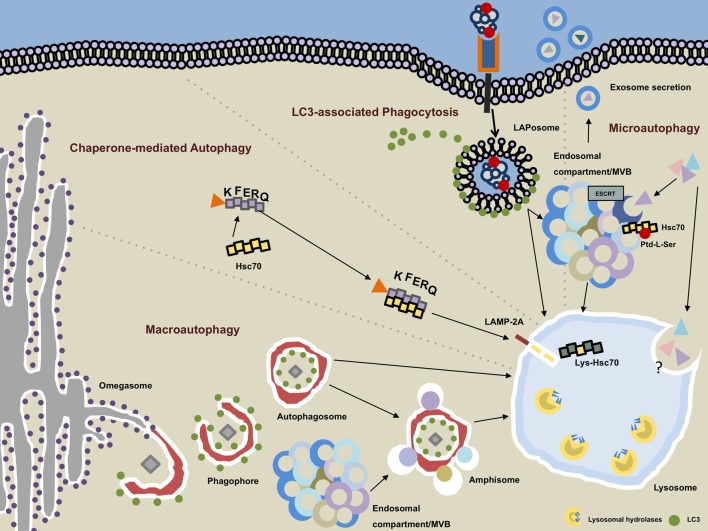
**Autophagy pathways converge in the lysosomal compartment**. Macroautophagy (MA): the phagophore emanates most likely from endoplasmic reticulum-derived membrane sources at a PI3P-rich (not depicted) structure called the omegasome. Upon recruitment of microtubule-associated protein 1 light chain 3 (LC3) on the outer and inner leaflet of the forming autophagosome, cytoplasmic cargo is engulfed and LC3 removed from the outer membrane as the autophagosome is closed. The completed vesicles may now either be further matured *via* step-wise fusion with the endocytic compartment resulting in the generation of amphisomes or immediately fuse with hydrolytic enzymes-containing lysosomes. Chaperone-mediated autophagy: target proteins that contain an exposed KFERQ sequence motif are guided in an hsc70-dependent manner toward lysosome-associated membrane protein type 2A (LAMP-2A), which resides in the lysosomal membrane. Upon unfolding of the target protein, LAMP-2A multimers together with lysosomal hsc70 facilitate the transport into the lysosomal lumen. LC3-associated phagocytosis: ligation of an appropriate receptor (e.g., TLR2, Dectin-1, TIM4, etc.) leads to receptor-mediated phagocytosis recruitment and binding of LC3 to the outer membrane of the LAPosome. By analogy with the MA pathway, completed LAPosomes may either fuse with other endocytic vesicles or directly merge with lysosomes. Microautophagy: cytoplasmic cargo can be directly targeted to endosomal compartments/multivesicular bodies (MVB) in an hsc70-, phosphatidylserine (Ptd-L-Ser)-, and endosomal sorting complexes required for transport (ESCRT)-dependent manner. Exosomes containing cytoplasmic material may emanate from the MVB and be secreted into the extracellular space. The molecular events and regulatory processes orchestrating the direct invagination of cytoplasmic constituents into lysosomes remain largely unbeknownst.

Macroautophagy, the canonical autophagy pathway *sensu strictu*, is evolutionary conserved from yeast to mammalian cells and characterized by highly regulated membrane reorganization processes with the subsequent *de novo* formation of a 0.5–1.5 µm wide double-membraned vesicle termed autophagosome (17). Upon sequestering neighboring parts of the cytoplasm, the autophagosome subsequently fuses with lysosomes resulting in enzymatic cargo break down (18, 19). The process is partitioned in five sequential steps (1. induction/nucleation, 2. elongation, 3. closing/maturation, 4. fusion, 5. degradation) that are orchestrated by hierarchies of autophagy-related genes/proteins (Atgs) and other essential components, in a tightly regulated enzymatic cascade (16, 20, 21) (Figure 1). Albeit originally identified in yeast, mammalian counterparts for many Atgs have been characterized and some Atgs are so far exclusively reported in mammalian cells and lack yeast orthologs (21). Among all autophagy pathways, MA is to date the most extensively investigated one and, depending on the target constituent encompasses subentities such as macromitophagy, -pexophagy, -xenophagy, and -lipophagy (21–25).

### Atg Machinery and Autophagosome Formation

*De novo* synthesis and maturation of the autophagosome as well as the trafficking of such vesicles to and fusion with lysosomes are distinctive features of MA in opposition to other autophagy pathways. Formation of this typifying vesicle requires approximately 5–10 min and is under the control of an ever-growing number of Atgs (19, 21, 26, 27). The finalized autophagosome is usually swiftly turned over but may reach a half-life of 10–25 min (28, 29). The key proteins that initiate and govern the formation of the autophagosome can be assembled in functionally designated groups: unc 51-like kinase (ULK) complex (1), the class III phosphatidylinositide 3-kinase (PI3K) complex (2), the Atg2/WD repeat domain phosphoinositide-interacting protein (WIPI) complex and the Atg9 cycling system (3), the Atg12-conjugation system (4), and the microtubule-associated protein 1 light chain 3 (LC3)-conjugation system (5) (19, 21, 30).

The autophagosome emanates from the double-membraned phagophore (also called isolation membrane), which sequesters and closes around designated parts of the cytoplasm to form the completed autophagosome. The emergence of said phagophore constitutes the induction/nucleation phase. However, the exact assembly platform and membrane source for the generation of these initial structures are still debated. Similar to the pre-autophagosomal structure that is observed adjacent to the vacuole in yeast, an autophagosome formation site, represented by dot-like accumulations of Atgs, has been identified in mammalian cells (31). Primary suspect organelles to provide membranes include specialized PI3P-enriched endoplasmic reticulum (ER) domains coined omegasomes (32). 3D electron tomography studies corroborated these results by showing that the double-membraned phagophore originates in between two protruding ER flaps. The phagophore then entwines one of the two extensions and finally buds off the ER containing the previously enfolded ER flap. This model is further supported by the fact that >70% of autophagosomes contain ER-derived cargo (33, 34). Nevertheless, other membrane sources for autophagosome generation have been suggested. Among them is the outer mitochondrial membrane (35). These two opposing results might be brought together by a recent study that identified ER-mitochondria contact sites as the originating platform for the autophagosome initiation (36). The VAMP3-dependent heterotypic fusion between early endosome-derived Atg9^+^ vesicles and recycling endosome-derived Atg16L1^+^ vesicles has also been suggested to contribute to autophagosome precursor generation (37). Additionally, ER exit sites (38, 39), the ER-Golgi intermediate compartment together with coat protein complex II (40, 41), the plasma membrane (42), and a novel compartment comprised of Atg9^+^ vesicles and tubules (43) were implicated in providing membrane material. It is conceivable that different subentities of MA preferentially harness distinct membrane sources. Possibly, there is a hierarchy of membrane reservoirs that sequentially may serve as alternative when other sources have been exploited. Furthermore, various tissues with specific composition of subcellular compartments may differ in their means to utilize membranes for autophagosome generation.

### The Atg Core Machinery

The ULK complex is an upstream Atg-unit and, by differential phosphorylation of ULK1 (the main mammalian ortholog to yeast Atg1), a direct target of MA regulation *via* target of rapamycin complex 1 (TORC1) and AMP-activated protein kinase (AMPK), respectively (44, 45). ULK1/2 builds a stable complex with FIP200, Atg13, and Atg101 (46–48). Not composition of the complex itself but rather differential phosphorylation of its members procures promotion or inhibition of MA. In a state of MA inactivation, TORC1 restrains the process by coordinate phosphorylation of ULK1/2 and Atg13. During activation, MA-promoting phosphorylation of ULK1/2 *via* AMPK, ULK1/2 autophosphorylation, and ULK1/2-mediated phosphorylation of Atg13 and FIP200 occur followed by translocation of the entire complex to autophagosomal initiation sites possibly on tubulovesicular areas comprised of ER and Atg9^+^ vesicles (46, 49–52). Atg101 may facilitate phosphorylation of Atg13 and minimize its proteasomal degradation (53, 54).

### The Class III PI3K Complex

The tetrameric core of this complex consists of vacuolar protein sorting protein (Vps)34 (a phosphoinositide 3 kinase), Vps15 (a regulatory subunit of Vps34 also called p150), Beclin 1 (ortholog of Atg6), and Atg14L (aka Atg14 and Barkor) (27, 55, 56). NRBF2 (ortholog of Atg38) is a recently identified additional subunit of the complex that augments the enzymatic activity of the lipid kinase Vps34 (57–59). The lipid kinase complex is recruited to autophagosomal initiation sites in an ULK1-complex/Atg9-dependent manner (31, 60).

The key function of the complex is to produce PI3P *via* Vps34-mediated phosphorylation of phosphatidylinositol. Presence and accumulation of PI3P are essential for MA as PI3P-enriched membrane areas then function as platforms to which downstream PI3P-binding partners can be recruited. Regulation of MA occurs also on the level of the PI3P complex in that death-associated protein kinase promotes autophagosome formation by unleashing Beclin 1 from the Bcl-2/Bcl-XL complex (61).

### Atg2/WIPI Complex and the Atg9 Cycling System

WIPI1/2 (mammalian ortholog to yeast Atg18) and its binding partner Atg2 are among the PI3P-binding effector molecules that are recruited to PI3P-enriched sites upon PI3PK complex activation (31, 62–64). Since PI3P is present on various subcellular membranes, up until recently it remained a conundrum as of how organelle-specific WIPI-binding is achieved. Studies in yeast have now suggested that WIPI-recruitment to the phagophore requires a dual determinant: PI3P as well as presence of Atg2 (65). The Atg2/WIPI complex is thought to be downstream of the two previous complexes but lateral to the following two conjugation systems (21). Its presence is essential for the formation of autophagosomes probably in part due to recruitment of lipidated LC3 (mammalian ortholog of yeast Atg8) to autophagosomal initiation sites and shielding of lipidated LC3 from Atg4-mediated deconjugation (66).

The transmembrane protein Atg9 can be found in the trans-Golgi network and on late endosomes under nutrient-rich (hence MA-inactive) conditions (67). Upon starvation (and induced MA activity) however, Atg9^+^ vesicles translocate from the trans-Golgi network and colocalize with LC3 (67). Possibly, Atg2 and WIPI1/2 mediate Atg9-shuttling between autophagosomal initiation sites and peripheral membrane sources (68). However, its interaction and association with the autophagosome appear to be transient since completed autophagosomes are devoid of Atg9 (69). It was also suggested that local fusion of Atg9^+^ vesicles operates as an initiation step in the generation of autophagosomes (43). Possibly, Atg9 functions in provision of membrane material for generation of autophagosomes or even removal of early autophagosomal molecules from the site of autophagosome generation (21). The exact interplay between the individual molecules of this unit and their overall function remains subject to further investigation.

### The Atg12-Conjugation System

The first of two ubiquitin-like conjugation systems that orchestrate autophagosome formation consists of the MA-essential molecules Atg12, Atg7, Atg10, Atg5, and Atg16L1 (21). Constitutively and independent of cellular nutrient status, Atg12 is activated *via* E1-like enzyme Atg7, followed by transfer to E2-like enzyme Atg10 which catalyzes the covalent conjugation of Atg12 to Atg5. *Via* binding to Atg5, the conjugate then forms a complex with Atg16L1 (70). WIPI2 now attracts the Atg5–Atg12–Atg16L1 complex by means of a recently identified binding site in Atg16L1 to the site of autophagosome generation (71). Additionally, it has been suggested that Atg16L1 is also recruited to the phagophore *via* binding to ULK-complex member FIP200 (72, 73). On site, the complex then functions as an E3-like enzyme for the second conjugation system. The complex is regularly found on the outer membrane of the phagophore but dissociates from there upon completion of the autophagosome (26).

### The LC3-Conjugation System

The second and final conjugation system is comprised of the ubiquitin-like LC3, the hydrolase Atg4, Atg7, and Atg3, which function as E1- and E2-like enzymes, respectively. The pro-form of LC3 is cleaved by Atg4 leading to exposure of a C-terminal glycine residue (74, 75). The resulting LC3-I is then lipidated with phosphatidylethanolamine (PE) at said glycine residue by means of Atg3 and Atg7 (76–78). This PE-lipidation of LC3 is assisted by the Atg5–Atg12-conjugates *via* E3-like activity (70, 79). Additionally, the Atg5–Atg12–Atg16L1-complex guides and determines LC3 to its subcellular destination (79). This lipidated form of LC3, also called LC3-II is initially found symmetrically distributed on the inner and outer membrane of the phagophore (75, 76, 80). There it appears to aid in elongation of the phagophore as well as coordinate tethering and hemifusion of its membranes and finally the closing of the phagophore to an autophagosome (81–84). Upon vesicle closure, LC3 on the outer membrane is being cleaved from PE *via* Atg4 while intraluminal LC3 stays associated with the organelle which makes LC3-II to this day a valuable marker for autophagosomes (80, 85). In addition to its contribution during membrane reorganization, LC3 mediates MA-cargo selectivity by functioning as an adaptor molecule (86).

## Regulation of MA

**Figure 2:**
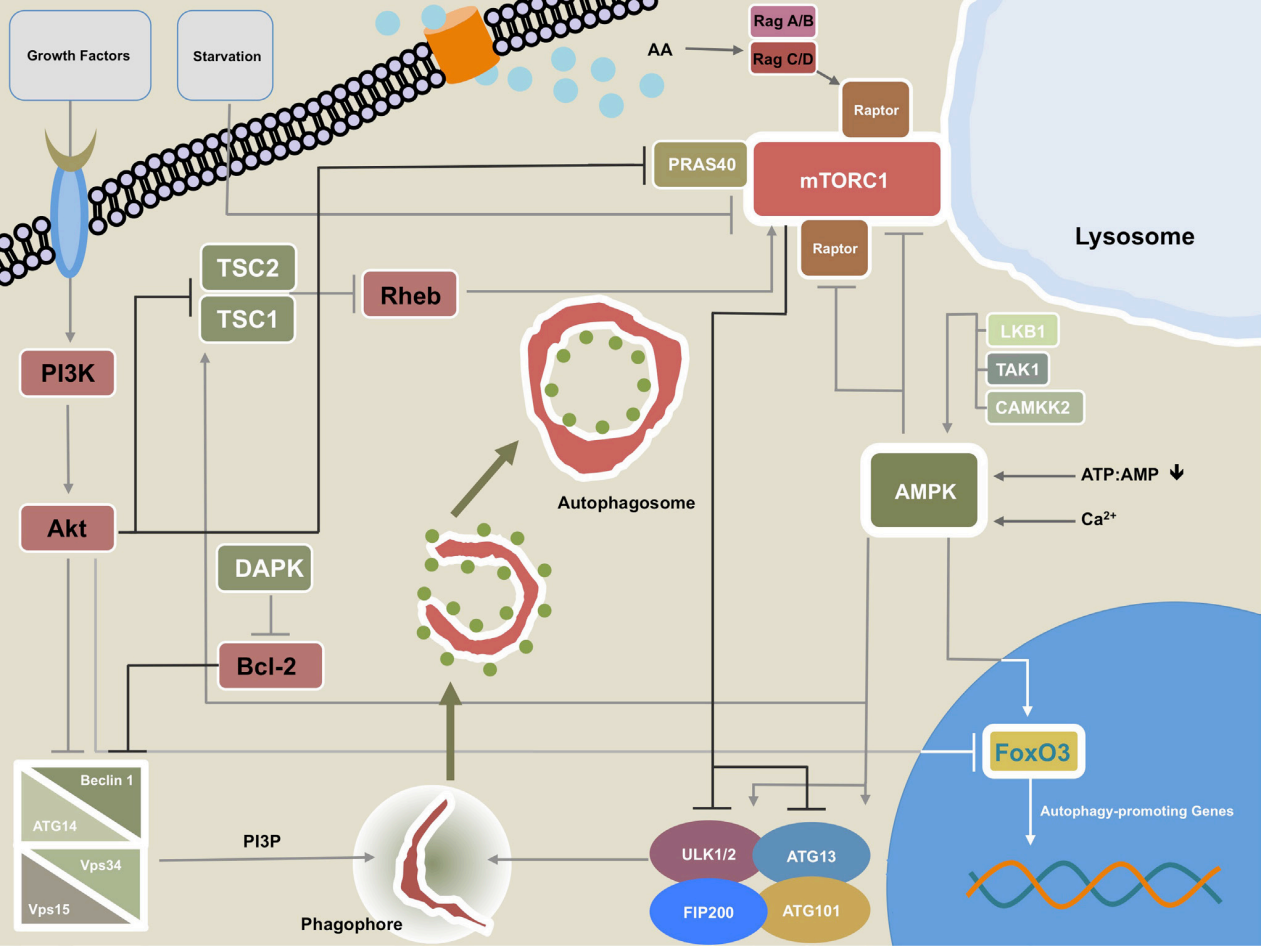
**Regulatory network of macroautophagy (MA)**. This figure illustrates the most important regulatory interplay of molecules that govern MA activity. Growth factors can signal through phosphatidylinositide 3-kinase (PI3K) to Akt, which in turn inhibits the Beclin 1-containing class III PI3K complex. Akt may also repress transcription of autophagy-promoting genes by direct inhibition of transcription factor forkhead box protein O3 (FoxO3). Additionally, Akt reduces macroautophagic activity by inhibiting tuberous sclerosis complex (TSC)1/TSC2. Akt interaction with PRAS40 promotes mTORC1 activity thereby further damping MA. The calcium/calmodulin (Ca^2+^/CaM) serine/threonine kinase death-associated protein kinase (DAPK) fosters MA through phosphorylation of Beclin 1 at T119 which leads to dissociation of Beclin 1 from Bcl-2. The TSC1/TSC2 complex can promote MA by inhibiting the mTORC1 activator and GTPase Ras homolog enriched in brain (Rheb). AMPK and mTORC1 are the two cardinal regulatory units that control MA. mTORC1 may repress the process by phosphorylation of ULK1/2 and/or Atg13. mTORC1 activity is increased by amino acid sensing Rag GTPases *via* interaction with Raptor. The Rag GTPases also facilitate translocation of mTORC1 to the lysosomal membrane, which serves as a prerequisite for the aforementioned mTORC1-promoting activity of Rheb. AMPK promotes MA by several means: phosphorylation of ULK1/2 and/or Atg13 at different residues than mTORC1, direct interaction with FoxO3, and by directly disinhibiting the inhibitory units, mTORC1 and Raptor. AMPK itself can be triggered by increasing levels of AMP relative to ATP, free Ca^2+^, and activity of liver kinase B1 (LKB1), transforming growth factor-β-activating kinase 1 (TAK1), and CAMKK2.

Most mammalian cells carry out MA on a constitutive level at varying degrees. However, depending on the cell type, macroautophagic activity can be induced and modulated in numerous ways. Primarily nutrient deprivation is a potent stimulus of autophagy and starvation-induced autophagy is a common means to investigate MA under experimental conditions (87, 88). The process receives regulatory input on both a systemic and a cellular level (88). Among the most upstream regulatory units of the MA machinery is the antagonistic interplay of coordinate phosphorylation by the two serine/threonine protein kinases AMPK and TORC1 (44, 45) (Figure 2). For insights into transcriptional and epigenetic regulation of MA, we kindly refer the reader to excellent recent review articles (89–91).

### mTOR/TORC1

Target of rapamycin complex 1 is one of two functional units of mTOR. Notably, as opposed to TORC2, which has been implicated as an inhibitory entity of CMA activity (92), TORC1 is actually a binding partner for the eponymous rapamycin and is the relevant subunit that participates in negatively regulating MA (93). Various signals that reflect a cell’s metabolic status (free amino acids, growth factors, fatty acids, oxygen, etc.) are integrated at the level of TORC1 which, during its activated state, restrains MA *via* phosphorylation of the upstream MA-machinery members ULK1/2 at serine 757 and ATG13 at serine 258 and is therefore considered a potent negative regulator of MA (46, 47, 94). In keeping with its cardinal function as a coordinator of metabolic homeostasis, two major inputs for TORC1 downstream signaling are free amino acids and growth factors (88). Growth factors signal *via* the PI3K–Akt–tuberous sclerosis complex (TSC)1/TSC2 pathway, eventually leading to disinhibition of the small GTPase Ras homolog enriched in brain (Rheb) which then directly activates TORC1 (95–97). Alternatively, the mTOR binding partner proline-rich Akt/PKB substrate 40 kDa (PRAS40) is directly phosphorylated by Akt which in turn leads to revoking of PRAS40-mediated inhibition of mTOR (98). Recently, Akt has been shown to also hamper MA in a direct manner *via* phosphorylation of Beclin 1 (99). Indirectly, Akt may repress MA *via* inhibiting forkhead box protein O3 (FoxO3) members-dependent transcription of autophagy-promoting genes (100–102).

Target of rapamycin complex 1 does not directly detect free amino acids and the actual amino acid sensing entity remains yet to be identified (88). Detection of free amino acids by innominate cytoplasmic sensors facilitates the Rag-GTPases-dependent translocation of TORC1 to the lysosomal membrane, which is a spatial requirement for Rheb-mediated activation of TORC1 (88, 103, 104).

### AMPK

AMPK is primarily an energy-sensing kinase that in response to metabolic stress and depleted cellular energy resources such as a low ATP:AMP ratio initiates catabolism (105). In doing so, AMPK is also a positive regulator of MA-mediated proteolysis (106). However, even under nutrient-rich conditions AMPK may act toward MA induction in a Ca^2+^-dependent manner (107, 108). Although essential for starvation-induced MA, AMPK might not suffice as a stand-alone signal but is likely to require additional cues in order to fully initiate MA (107, 108). Liver kinase B1, calcium/calmodulin-dependent protein kinase kinase-β (CAMKKβ), and transforming growth factor-β-activating kinase 1 are upstream regulators which through phosphorylating its catalytic α-subunit activate AMPK (109–113).

AMPK promotes MA by different pathways such as initiation of Rheb inhibition through TSC1/TSC2 (114), phosphorylation of the mTOR binding partner raptor, thereby inhibiting TORC1 (115), or direct phosphorylation of ULK1/2 (at serine 317 and serine 777) or Atg13 (at serine 224) which activates these family members of the upstream MA machinery (44, 45, 94). Furthermore, AMPK positively regulates MA through activation of the transcriptional factor FoxO3 which in turn transactivates MA-essential genes (116, 117).

## Microautophagy

During MI, a vaguely characterized autophagosome-independent process, superfluous cytoplasmic constituents including whole organelles are directly engulfed by surface invaginations of lyso- or endosomal surfaces leading to the budding of cargo-containing microvesicles into the endolysosomal lumina where degradation takes place (118–121) (Figure 1). MI initially has been termed unselective; however, numerous reports in yeast and mammalian cells challenged this concept and described more discerning mechanisms and functions underlying this pathway (118, 120, 122). For instance, the MI-assisted transport of endosomes to lysosomes appears to be a crucial event during murine embryogenesis in a Rab7-dependent manner (123). Endosomal sorting complexes required for transport (ESCRT)-machinery-dependent microlipophagy is essential during cellular adaptation to cell stress caused by disruption of lipidostasis in yeast (124). Schuck and colleagues characterized the specific uptake of compromised ER into the yeast vacuole and coined the term ERphagy (125). Other cargo selective MI entities in yeast include micromitophagy (126, 127), micropexophagy (128, 129), and piecemeal microautophagy of the nucleus (130–132). To which extent these processes take place in mammalian cells remains largely obscure (133). Little is known about MI and its regulation on a molecular level in mammalian cells and most findings rely on electron microscope-based morphological alterations such as lysosomal wrapping and flap-like lysosomal membrane extensions (133). The picture becomes even more hazy when acknowledging that some forms of MI require the core machinery needed for MA (see above) but others do not (120, 128, 132, 134). However, distinct cargo selective MI pathways have also been identified in mammalian cells. For instance, heat shock cognate protein of 70 kDa (hsc70)-dependent targeting of cytosolic KFERQ sequence motif-containing proteins into late endosomes and multivesicular bodies (MVB), termed endosomal MI, has recently been identified in murine antigen-presenting cells (APCs) (120). Although this newly characterized process resembles the selective pathway of CMA (see below) into lysosomes, this endosome/MVB-destined MI does not require unfolding of target proteins and is lysosome-associated membrane protein type 2A (LAMP-2A) independent (120). Also different from CMA, endosomal MI is not only dependent on hsc70 but also heavily relies on ESCRT I and III and involves electrostatic binding of hsc70 to negatively charged endosomal phosphatidylserine (Ptd-L-Ser) (120, 135). Fission yeast Nbr1, a partial homolog of mammalian MA receptor NBR1, has been recently identified as a receptor for ESCRT-dependent endosomal MI (136, 137). Two recent reports further elaborated on this novel pathway. Mukherjee and colleagues describe an endosomal MI-like process in the fat body of *Drosophila melanogaster*. In accordance with what had been reported previously, this process is KFERQ- and hsc70-dependent and relies on ESCRT I-, II-, and III-mediated endosomal MVB formation. Although this pathway is independent of MA-machinery members—*atg5, atg7*, and *atg12*—it can be induced by prolonged starvation and by mTOR signaling under nutrient-rich conditions (138). A second report shows that by means of its oligomerization capabilities, hsc70 promotes membrane deformations, which supports presynaptic endosomal MI and increases the local turnover of target proteins. Importantly, the degree of presynaptic endosomal MI fosters the release of neurotransmitters into the synapse (139).

Intraluminal vesicles generated within MVBs give rise to exosomes which in a controlled and coordinated manner are released to the extracellular space by most cells (140, 141). Aside from their physiological function in cell-to-cell communication and coordinating immune responses, these vesicles of endocytic origin have also been implicated in propagating neurodegenerative processes (141–143). Conclusive evidence points toward a substantial role of endosomal MI in providing cargo for this unique secretory pathway (120, 141). It will be of great importance to elucidate the mechanisms and triggers that decide whether MVB content is targeted toward intracellular degradation by the lysosome or instead is fed into the secretory pathway *via* exosomes.

## Chaperone-Mediated Autophagy

Chaperone-mediated autophagy is a selective degradation process devoid of autophagosome formation during which substrate proteins are directly targeted to the lysosome (144) (Figure 1). There is no known equivalent in yeast and evolutionary speaking CMA has emerged only recently, thus far exclusively being identified in mammals (145). Most cells constitutively carry out CMA but the process is upregulated in response to oxidative stress (146), hypoxia (147), DNA damage (148), or protracted nutrient deprivation (149). Unlike MI and MA, which may also handle surplus organelles and non-protein macromolecules, CMA exclusively processes proteins that contain a pentapeptide motif in their amino acid sequence (KFERQ) (150). CMA encompasses at least five distinct steps (1. recognition, 2. binding, 3. unfolding, 4. translocation, 5. disassembly) (144): target proteins bind with their KFERQ-recognition motif to the cytosolic chaperone hsc70 and the substrate:chaperone complex translocates to the lysosomal membrane (recognition) (151). There, LAMP-2A, a CMA-receptor, binds the substrate:chaperone complex *via* its cytosolic tail and subsequently the designated protein undergoes unfolding in a hsc70-dependent manner probably assisted by co-chaperons on the cytosolic side of the lysosome (binding and unfolding) (152, 153). Binding of the substrate:chaperone complex to monomeric LAMP-2A not only initiates protein unfolding but also recruitment of further LAMP-2A molecules and subsequent assembly of high molecular weight multimers that then, by help of lysosomal hsc70 (lys-hsc70) enable passage of target proteins to the luminal side of the lysosome (translocation) (154). Upon release of the target protein into the lysosomal lumen, the LAMP-2A multimers are hsp70-dependently broken down into monomers which are then available for another cycle of ligand binding and uptake (disassembly) (154). Recently, more light has been shed on the molecular regulatory pathways controlling CMA. Glial fibrillary acidic protein (GFAP) together with elongation factor 1α tunes assembly and disassembly of the LAMP-2A translocation complexes at the lysosomal membrane (155). Phosphorylation of GFAP by lysosomal Akt1 leads to destabilization of such translocation complexes and consequently reduced CMA activity. The antagonistic interplay between PH domain leucine-rich repeat-containing protein phosphatase 1 (PHLPP1) as a negative and mTORC2 as a positive regulator upstream of lysosomal Akt1 signaling therefore appears to play a central role in modulating CMA activity (92). Different from MI and MA, CMA processes one molecule at the time and does not require morphological changes of lysosomal membranes such as invaginations, wrapping, flap-like extensions, or fusion with other membranes (156, 157). The main rate limiting factor in CMA is abluminal LAMP-2A availability which is regulated by the degree of LAMP-2A degradation by a metalloprotease and cathepsin A at the site of designated lipid microdomains in the lysosomal membrane (158). As previously mentioned, a prerequisite for CMA processing of a target protein is the presence of an amino acid sequence motif biochemically related to KFERQ (150). In contrast to the biochemical earmarking of a molecule by means of ubiquitination, which may designate the target for proteasomal degradation or recognition by ubiquitin-recognizing adaptor molecules, the KFERQ consensus motif is pre-existing in CMA-target proteins (145). It is therefore likely that the pentapeptide motif is not ubiquitously accessible for binding to hsc70 in order to prevent premature protein translocation to the lysosomal lumen *via* CMA. Possible mechanisms to condition a target for this destination include but are not limited to, partial unfolding, disassembly of multimers, cleavage of proteins, and posttranslational modifications such as phosphorylation or acetylation of amino acid residues (145, 159, 160). Cytosolic accumulation of aberrant proteins is a hallmark of age-related proteinopathies of the brain and other tissues. A central function of CMA is the removal of misfolded or unwanted proteins and dysfunction in CMA in senescent cells has been implicated in promoting neurodegeneration (161–163). Not considering posttranslational alterations, roughly 30% of cytosolic proteins bear a sequence motif biochemically related to KFERQ and are therefore potentially subject to CMA-mediated lysosomal degradation (145, 150, 151). Due to the heterogenous nature of this protein pool it does not come as a surprise that CMA can be regarded as a pivotal gatekeeper in energy homeostasis that also fine-tunes vital cellular processes by limiting key metabolic enzymes (144).

## Autophagosome Maturation and Fusion with the Endolysosomal System

A hallmark of all autophagic pathways is their convergence into the lysosomal system. During MA, this event requires the coordinated membrane fusion of the autophagosome and endolysosomal vesicles (Figure 1). Prior to terminal fusion with lysosomes, autophagosomes may (or may not) fuse with vesicles of the endocytic compartments like early or late endosomes. The resulting amphisomes subsequently fuse with lysosomes to form autolysosomes. These partly sequential, partly parallel fusion events underscore the dynamic nature of MA. Hence, observed accumulation of autophagosomes needs to be carefully interpreted, for it can mean both *bona fide* induction of the process and blocked lysosomal fusion.

Autophagosomes are widely distributed throughout the cytoplasm. For these vesicles to fuse with lysosomes and late endosomes (which are predominantly located juxtanuclear), autophagosomes need to be efficiently guided toward this area. Members of the cytoskeleton have been shown to orchestrate the regulated trafficking of autophagosomes from the periphery to sites at which membrane fusion occurs. In fact, microtubules might even aid in autophagosome formation and subsequent fusion with endosomal compartments (164, 165). In an antagonistic interplay, the members of the motor protein family dynein/dynactin complex and kinesin have shown to be involved in guiding autophagosomes alongside microtubules toward lysosome-rich areas, a process during which the trafficked vesicles become increasingly acidified as they approach the juxtanuclear area (166, 167). Consequently, disruption of the dynein machinery exacerbates aberrant protein aggregation in experimental models of neurodegeneration due to dysfunctional MA (168). Interestingly, dynamic distribution of lysosomes within the cytoplasm may actually constitute a mechanism by which autophagic flux is regulated (169).

In accordance with general membrane reorganization, also the fusion of autophagosomes with endolysosomal vesicles is in large parts orchestrated by members of small GTPases called Rabs, membrane-tethering complexes, and SNAREs. The molecular specifics have been reviewed in detail elsewhere (170, 171). Studies in yeast suggest that Atg4-mediated cleavage of the LC3 ortholog Atg8 from the outer membrane constitutes one prerequisite that renders the autophagosome ready for fusion (172, 173). Possibly, absence of molecules that are involved in the early phase of autophagosome biogenesis functions as yet another signal (170).

Our understanding of the precise molecular events during autophagosome biogenesis and fusion has significantly improved during the last decade and enticing models have been proposed as on how the phagophore is initiated. However, one needs to be cautious as of how the aforementioned molecular interplay of Atgs can be generalized since most results were obtained studying starvation-induced MA. There is evidence that autophagosomal biogenesis, and subsequent fusion partners, -sites, and -mechanisms are highly dependent on induction stimulus and cargo (170). Furthermore, the degree as to which these processes are actually carried out in sequence remains enigmatic. It is likely that numerous steps occur in parallel and distinct Atgs might not only carry out a single function but take on several tasks within the cascade. The molecular interactions between Atgs and the kinetics of the process might also significantly differ within the mammalian species and even on a tissue level, there might be specifics to MA that need to be taken into account.

Finally, at least for some Atgs that are essential for MA, an even more promiscuous role has begun to unfold in that these proteins also facilitate MA-independent functions in cellular reprograming, dynamic membrane re-distribution, pathogen clearance, and antigen presentation (11, 174–178).

## Non-Canonical Autophagy Pathways

Non-canonical autophagy comprises a set of recently characterized pathways that either result in autophagosome formation but omit the usage of distinct parts of the classical MA-machinery or autophagosome-independent pathways that utilize key components of the MA network (16, 179). One can at least differentiate five distinct entities: *LC3-associated phagocytosis (LAP), Beclin 1-independent autophagy, autophagosome formation from multiple phagophores and pathogen-specific autophagy modification, autophagy-associated unconventional protein secretion*, and *defective ribosomal products-containing autophagosome-rich blebs*. Here, we focus on LAP, since this pathway has been associated with antigen processing and presentation, and kindly refer the reader for specifics on the other non-canonical autophagy pathways to excellent reviews elsewhere (16, 179, 180).

### LC3-Associated Phagocytosis

**Figure 3:**
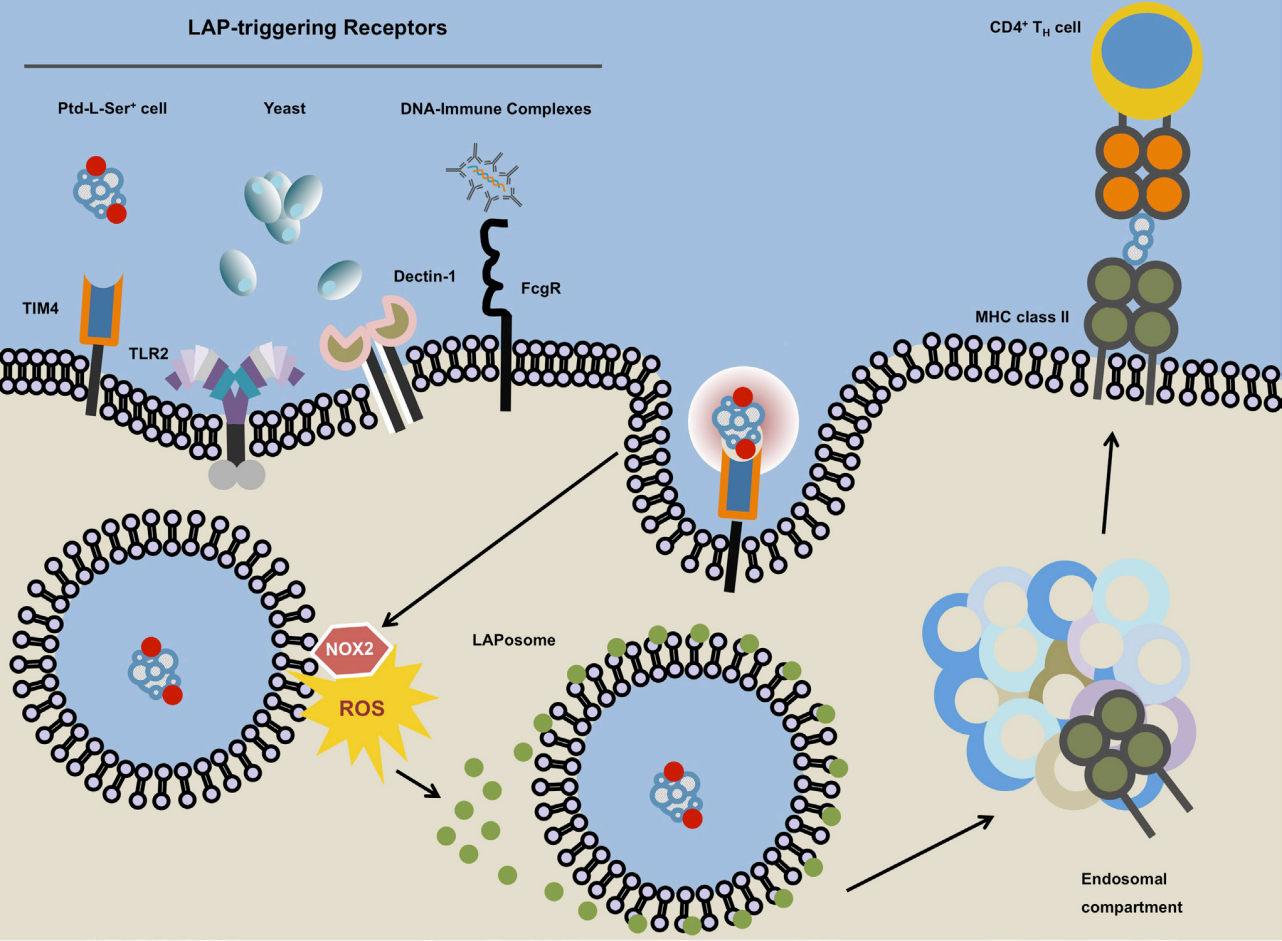
**Microtubule-associated protein 1 light chain 3 (LC3)-associated phagocytosis**. The ligation of LC3-associated phagocytosis (LAP)-triggering receptors such as phosphatidylserine (Ptd-L-Ser)-recognizing TIM4, TLR2, Dectin-1, or Fcγ receptors will ensue receptor-mediated phagocytosis and subsequent phosphatidylinositide 3-kinase-dependent association of the NADPH oxidase NOX2 with the phagosome. The NOX2-derived reactive oxygen species (ROS) and laposomal PI3P (not depicted) mediate the recruitment and binding of LC3 to the outer membrane of the LAPosome. Instead of terminal fusion with the lysosomal compartment, the completed LAPosome may also fuse with endosomal vesicles including major histocompatibility complex (MHC) class II-containing compartments (MIICs) in which after enzymatic digestion, LAPosomal content can be loaded upon MHC class II molecules followed by the presentation of the resulting peptides to T_H_ cells.

During phagocytosis, a specialized way of endocytosis, cells internalize solid extracellular constituents in a receptor-mediated fashion. The resulting phagosome is being step-wise matured and subsequently fuses with the lysosomal compartment in order to break down the incorporated material (181). Recently, a novel organelle, the single-membraned LC3^+^ phagosome or LAPosome, has been identified and the process of its generation and fate was coined LAP (Figure 3) (182, 183). LAP, that links both phagocytosis of extracellular cargo and members of the autophagy molecular core machinery, is initiated by ligation of a variety of extracellular receptors (12, 182, 184–186). Although some components of the MA machinery are essential for LAP (e.g., Atg5, Atg7, LC3, Beclin 1), the process is independent of others (e.g., the ULK complex including ULK1/2, FIP200, Atg13, Atg101, WIPI1).

LC3-associated phagocytosis-triggering receptors include toll-like receptors (TLRs), Fc-receptors, C-type lectins, and Ptd-L-Ser-binding receptors (12, 182, 184–186). Upon activation of a LAP-triggering surface receptor, a PI3PK complex that differs in its composition from the one involved in MA, is recruited to the cytosolic membrane of the phagosome followed by the recruitment of the NADPH oxidase NOX2. These events are not preceded by involvement of the canonical ULK complex. The LAP-associated PI3PK complex is made up by Beclin 1, Vps34, and Vps15 (analogous to MA) but is devoid of Atg14 (187). Instead, it includes UVRAG and Rubicon (not members of the canonical PI3PK complex). Similar to MA, the PI3PK complex is set out to generate PI3P on the LAPosome. The association of the modified PI3PK complex on the LAPosome is potentially liaised *via* Rubicon which, together with PI3P also appears to be key in recruiting, stabilizing, and activating the NADPH oxidase NOX2 to the organelle (187). Rubicon mediates NOX2 stabilization by interaction of its serine-rich domain (AA 567–625) with the NOX2 subunit p22^phox^ whereas PI3P stabilizes the NOX2 subunit p40^phox^ (187, 188). LAPosomal PI3P in concert with NOX2-dependent reactive oxygen species (ROS) production then initiates the two canonical MA conjugation systems (see above), which results in efficient deposition of lipidated LC3 on the outer LAPosomal membrane (187). Consequently, LAP is highly dependent on the MA proteins Atg5, Atg12, Atg16L1, Atg7, Atg3, Atg4, and lipidated LC3. Molecules that have been described to be dispensable for canonical MA but are thought to be essential for LAP include NOX2 and Rubicon (187).

Taken together and in contrast to the canonical MA pathway, during which LC3 conjugation to the phagophore allows for recruitment of cytoplasmic substrates into forming autophagosomes by means of LC3-binding anchor proteins such as p62/sequestosome 1, LAP handles formerly extracellular particles that access the cell through phagocytosis and occurs without the formation of a double-membraned vesicle and in the absence of p62/sequestosome 1 on LC3^+^ single-membraned phagosomes (179, 183, 187).

So far LAP has been implicated in efficient clearance of *Saccharomyces cerevisiae* and *Aspergillus fumigatus* (182, 187). However, in the case of *Listeria monocytogenes* infection, LAPosomes have been described as *Listeria-*containing compartments that promote persistent infection (189). In plasmacytoid dendritic cells (DCs), LAP, induced upon binding of immune complexes through FcγR, is required to assemble the interferon regulatory factor 7 (IRF7)-signaling compartment which is essential for IRF7 activation and subsequent IFNα secretion downstream of TLR9 ligation (186). LysM-Cre^+^ conditional knockout mice for LAP-essential proteins mainly target CD11b^+^/F4/80^+^ macrophages and CD11b^+^Ly6G^+^ neutrophils. Aged mice that are deficient of LAP activity in these myeloid subsets spontaneously acquire a lupus-like phenotype (190).

Several questions remain unanswered. The functional benefit of coupling lipidated LC3 to a phagosome is still not completely understood. Some have argued that the coating of the phagosome with LC3-II allows the vesicle to travel faster along microtubules which results in faster fusion with the lysosomal compartment (182, 185, 191). However, studies that report more rapid cargo degradation were predominantly obtained using murine macrophages. Human studies on the other hand show that in prototypical APCs such as DCs, LAP seems to retain antigenic cargo for sustained MHC class II antigen presentation rather than promoting its degradation (12). Antigenic containment and stabilization in combination with low levels of lysosomal proteases have been speculated to be a crucial mechanism by which DCs maintain efficient and prolonged antigen presentation (12, 183, 192). Lee and colleagues reported that in murine DCs, TLR-dependent phagocytosed HSV-2 can be found in LC3^+^ single-membraned vesicles reminiscent of LAPosomes. In absence of LAP-essential protein Atg5, subsequent HSV-2-specific CD4^+^ T cell responses were markedly reduced, arguing for inefficient MHC class II-dependent presentation of LAP-deficient DCs toward cognate T cells (193). Interestingly, pharmacological induction of canonical MA by TORC1 inhibitor rapamycin did not lead to enhanced MHC class II presentation of viral antigens arguing for a non-MA pathway linking phagocytosis and MHC class II presentation (193).

## Autophagy Pathways in CNS Autoimmunity

**Figure 4:**
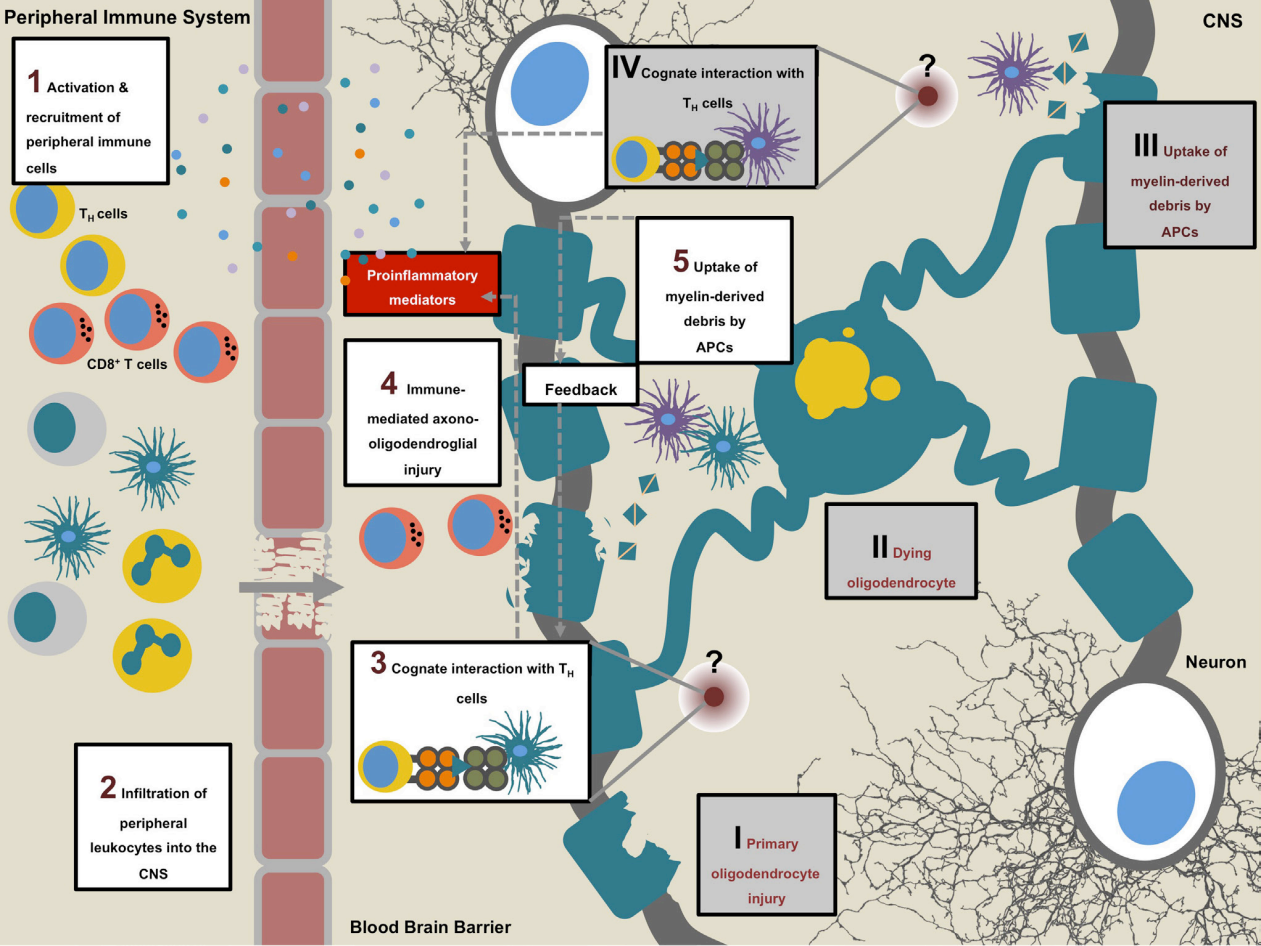
**Multiple sclerosis (MS) pathogenesis**. Sequence of events that initiates the onset of disease (1–5). MS is suspected to be an autoimmune condition during which peripheral autoaggressive T cells cross into the central nervous system (CNS) where, in concert with other leukocytes, they mediate axono-oligodendroglial damage and unleash a circle of self-perpetuating neuroinflammation (1–5). An alternative to this “outside-in” sequence is the “inside-out” model, which proposes a primary disease triggering event within the CNS (I–IV): primary oligodendrocyte damage of unknown cause may lead to dying and disintegrating oligodendrocytes followed by the uptake of myelin-derived material by CNS-resident antigen-presenting cells (APCs). Upon further intracellular processing of the phagocytosed material, APCs present antigenic myelin-derived peptides to T_H_ cells (at an as of yet unknown site) which in turn initiate influx of peripheral immune cells by secretion of proinflammatory cytokines and chemokines. On site, infiltrating immune cells, including cytotoxic CD8^+^ T cells and myeloid cells perpetrate secondary oligodendrocyte damage.

Multiple sclerosis is a chronic neuroinflammatory condition during which autoaggressive leukocytes invade across a compromised blood–brain barrier into the CNS. On site, the infiltrated immune cells, in concert with CNS-resident cells mediate progressive axonal demyelination and engender spatio-temporally disseminated lesions within the CNS which entails diffuse cytodegeneration in gray and white matter areas of the CNS (194) (Figure 4).

Experimental autoimmune encephalomyelitis (EAE) is a commonly used animal model of MS in which disease is induced by immunization of animals with myelin-derived antigenic peptides together with adjuvant or by adoptively transferring pre-activated myelin-specific CD4^+^ T cells in naïve recipients (195–197). EAE is a predominantly T cell-driven model and a pathomechanistic prerequisite for CNS damage is the local reactivation of CD4^+^ T cells specific for myelin-derived antigens (198–202). Reactivated CD4^+^ T cells together with activated myeloid cells initiate a cascade of proinflammatory events, which is believed to perpetuate CNS tissue damage leading to the development of clinical EAE symptoms (203–205).

How and by which APC subset pathogenic CD4^+^ T cells are being locally reactivated and cause tissue damage during the course of MS and EAE is, however, incompletely understood. This is in part due to the fact that the detailed antigen-processing route of how myelin self-antigens are processed and presented toward CD4^+^ T cells has not been fully elucidated.

Given their diverse, yet often essential functions in immune surveillance, cell survival, proteostasis, antigen presentation, and regulating cellular energy levels, a role for autophagy pathways in the complex pathobiology of MS can been proposed in many aspects of the condition including but not limited to the innate and adaptive branches of the immune system. In the following sections, we will discuss how autophagy pathways potentially interfere with pathogenic adaptive immune responses in MS.

## Autophagy in T Cells

Autophagy has a central but complex role in cell survival in numerous cell types, functioning either as a pro-survival or as a cell death mechanism depending on the cell type, the nature of the death stimulus, and subsequent compensatory changes. Atg5-deficient CD4^+^ and CD8^+^ T cells develop normally in the thymus, but fail to repopulate the periphery because of massive cell death and fail to undergo efficient proliferation after T cell receptor stimulation (206). Using a T cell-specific conditional knockout of Beclin 1 (CD4-Cre Beclin 1^fl/fl^), Kovacs et al. later elaborated on the underlying mechanism and confirmed that in absence of Beclin 1 in the CD4^+^ CD8^+^ T cell compartment, homeostasis was disturbed. In line with previous reports, thymic development of the T cell compartment was normal; however, numbers of CD8^+^ T cells in secondary lymphoid organs were drastically diminished (>3-fold), whereas CD4^+^ T cell were less affected (reduction of CD4^+^ T cell numbers amounted to <2-fold). The steady state fractions of naïve and memory T cells were unchanged between conditional KO mice and controls. Upon activation with anti-CD3/anti-CD28, however, T cells readily underwent apoptosis. Increased susceptibility to cell death was linked to MA-dependent degradation of pro-apoptotic proteins and seems to be differentially relevant in CD4^+^ T cell subsets (207).

Additionally, Atg16L1-specific deletion in T cells results in spontaneous intestinal inflammation and inbalances of different T_H_ cell subsets in the gut. While intestinal T regulatory cells (TREGs) are drastically reduced in number, IL-13-producing T_H_2 cells expand (208). TREG-specific deletion of Atg5 and Atg7 entailed reduced frequencies and survival of this subset and leads to defective self-tolerance (209).

These data indicate a critical role for the autophagy machinery in lymphocyte development and function and suggest that MA may be essential for both T lymphocyte survival and proliferation in the steady state and after immune activation.

In line with these assumptions, CD4-Cre Beclin 1^fl/fl^ mice are completely protected from active induction of EAE (207). Interestingly, the defect in the CD4^+^ T cell compartment during EAE is much more pronounced in the T_H_1 fraction but only mildly present in T_H_17. Subsequent *in vitro* experiments further confirmed that T_H_1 cells are significantly more prone to undergo apoptosis in absence of autophagy (207).

In addition to the observed protection of EAE upon T cell-specific ablation of autophagy, Atg5 transcript and protein levels in circulating T cells positively correlated with the disease score in mice that had been actively immunized with MOG_35–55_ peptide. Furthermore, peripheral T cell-derived RNA analyses suggested a significant increase in Atg5 expression in patients with active relapsing-remitting MS as compared to healthy controls. Atg5 RNA levels in postmortem brain tissue from patients with secondary progressive MS (SPMS) were markedly elevated and strong Atg5 immunoreactivity was observed in confocal microscopy analyses (210).

In contrast to what Kovacs and colleagues had reported, another study that used Beclin 1-deficient Rag1^−/−^ chimeras, found that although Beclin 1 is essential for the maintenance of early lymphocyte progenitor subsets, it is widely expendable for the homeostasis and function of peripheral T and B cells (207, 211). Differential efficiencies of genetic targeting and overall differences in the genetic ablation models may partly account for these discrepancies.

Recently, an additional role for autophagy proteins in the maintenance of CD8^+^ memory T cells has emerged. Mice that lack Atg7 in T cells showed reduced CD8^+^ T cell-dependent recall responses upon infection with influenza and MCMV (212). Impairment of CD8^+^ T cell memory in elderly individuals is commonly observed and in part responsible for the reduced efficacy of vaccination with aging (213). Interestingly, Puleston et al. also found reduced levels of autophagic activity in CD8^+^ cells of aged mice. Accordingly, upregulation of autophagy with the polyamine spermidine could restore memory CD8^+^ T cell function to vaccination in aged mice (212). In contrast to what Pua and colleagues had reported previously (206), another study described how CD8^+^ cells downregulate autophagic activity upon activation and during proliferation. This was followed by increased activity before the contraction phase. Absence of the autophagy proteins Atg5 and Atg7 lead to impaired formation of a CD8^+^ T cell memory pool. Moreover, metabolomics analysis disclosed mitochondrial fatty acid oxidation to be dysfunctional in Atg7-deficient T cells at the transition to the memory phase (214).

Aside from the role of autophagy in T cell survival and homeostasis, also CMA has been implicated in regulating CD4^+^ T cell responses. The negative regulators of TCR signaling, RCAN1, and Itch have been identified as substrates for CMA. Thereby, CMA-controlled degradation of these molecules fine-tunes T cell responses which is exemplified by the significant reduction in activation-induced proliferation of CD4^+^ T cells that lack LAMP-2A expression (215). Although so far, dysfunctional CMA in T cell has not been formally reported to occur in MS or its model systems it is known that mice lacking Itch are prone to develop a hyperactive T cell-driven systemic progressive autoimmune condition (216).

Similar to T cell development, B cells require autophagy both during development and maintenance in the periphery. Atg5-deficient pro-B cells do not efficiently develop into pre-B cells, but instead, seem to die at increased frequencies of apoptosis (217). Altogether, these data suggest that highly proliferative lymphocyte compartments during development or in response to immune activation, as seen in autoimmune CNS conditions, require the autophagic machinery to efficiently mobilize nutrients and maintain cellular fitness. However, further studies are needed to clarify differential roles of autophagy pathways in lymphocyte subsets particularly in the context of T cell-driven autoimmunity.

## Autophagy and Antigen Presentation

Similar to other autoimmune diseases, HLA-DR and -DQ alleles within the HLA class II region on chromosome 6p21 are by far the strongest risk-conferring genes and are thought to account for 10–60% of the genetic risk of MS (218). The size of the autoreactive T cell pool is limited by mechanisms of tolerance; however, it is clear that potentially dangerous cells persist in the immune system of healthy individuals without causing damage. Although it is generally accepted that HLA class II molecules influence autoimmune disease risk by regulating the emergence, activation, and expansion of autoreactive CD4^+^ T lymphocytes, our knowledge of how HLA class II-mediated antigen presentation confers risk for autoimmune diseases and regulates CD4^+^ T cell autoreactivity at the molecular level is incompletely understood (219, 220).

The cellular architecture of MS lesions is dynamic in its neuropathological features including leukocyte composition (221). Commonly, T cell infiltration occurs in two distinct waves (222, 223). Initially, MS lesions are characterized by oligodendrocyte damage and commencing demyelination accompanied by microglia that show an activated phenotype. However, in these initial lesions lymphocytes are scarce. Thereupon progressive demyelination occurs, and myelin constituents are phagocytosed by microglia and other myeloid cells which ensues a dramatic T cell influx (224).

Onset of EAE is fully dependent on the presence of myelin-specific T_H_ cells and local reactivation of these pathogenic culprits is essential in developing CNS autoimmunity (198–202). To this effect, on-site presence and activity of CD11c^+^ APCs is an instrumental prerequisite for disease induction during the effector phase of EAE (199). Moreover, intravital 2-photon microscopy analyses revealed that during EAE, CD11c^+^ APCs show a strong proinflammatory phenotype, express a chemokine profile complementary to the chemokine receptor profile expressed on encephalitogenic T cells in the CNS and depict close spatial interactions with these lymphocytes. Hence, depletion of CD11c^+^ APCs abrogated enrichment of pathogenic T cells in the CNS and significantly alleviated disease course upon adoptive transfer EAE (225). Active MOG_35–55_ immunization of mice that lack expression of Atg7 in the CD11c^+^ compartment results in ameliorated EAE disease course (226). Therefore, further elucidation of the underlying molecular events that drive these cognate interactions between the triad of (CNS resident) APC, myelin antigen, and encephalitogenic T cell, might unveil new potential therapeutic objectives.

The classical MHC class II pathway, the MA pathway, and LAP are potential degradation pathways resulting in MHC class II presentation of self-antigens. The first compelling evidence for the existence of an endogenous MHC class II pathway came from a study on describing how the endogenous measles virus matrix and nucleocapsid protein-derived antigens could be presented to CD4^+^ T cells *via* MHC class II (227). Only 1 year later, Nuchtern et al. confirmed these findings for yet another viral antigen by reporting the efficient loading of influenza A matrix protein-derived peptides onto MHC class II (228). Further proof for the existence of endogenous MHC class II loading pathways was obtained through the direct analysis of peptides bound to MHC class II molecules. In fact, around 30% of natural ligands eluted from MHC class II molecules from a multitude of cell types were found to be derived from endogenous protein sources (229). In a next step, MA was newly identified as a pathway that delivers endogenous antigenic constituents into MIICs for subsequent recognition by CD4^+^ T cells (9, 10, 230). Interestingly, the ability to deliver endogenous antigens to MHC class II seems to be differentially relevant depending on the APC (231). MA is constitutively active in a variety of MHC class II positive APCs such as DCs and B cells (9, 230, 232) and can be induced following immune stimulation of these cells through germline-encoded pattern-recognition receptors such as TLRs or inflammatory cytokines (233–235). Co-localization studies indicate that in professional APCs, autophagosomes frequently fuse with MIICs, and that experimental delivery of antigens to autophagosomes by targeting the autophagosomal membrane through fusion with LC3 results in robust recognition by antigen-specific CD4^+^ T cells (10, 232). Additionally, not only hematopoietic APCs but also thymic epithelial cells expend MA to generate endogenously derived MHC class II-bound peptides for positive selection of CD4^+^ T cells (236).

Nevertheless, myelin-derived candidate antigens are not known to be expressed in professional APCs therefore an intracellular loading of these peptides onto MHC class II molecules in the context of CNS autoimmunity appears counterintuitive. LAP couples phagocytosis of extracellular solid cargo to key members of the autophagy machinery (179, 187). A prerequisite for LAP is the engagement of germline-encoded pattern-recognition receptors or Ptd-L-Ser receptors through phagocytosed material. The role of TLR signaling in the context of EAE has partly been investigated. TLR9 and, to some extend, TLR2 signaling mediates the pathogenicity of EAE, whereas TLR1- and 6-deficiency in mice did not have an impact on the development of active EAE (237, 238). Importantly, also adoptive transfer EAE-induction was dependent on TLR2 signaling, discarding the assumption that microbial-derived constituents in the adjuvant preparation during active EAE serve as ligands (238). Additionally, both studies showed that deficiency of the downstream TLR-signaling molecule myeloid differentiation primary response gene 88 (MyD88) abrogates disease (237, 238).

Ample data demonstrate that oligodendrocyte injury and death represented by primary demyelination in absence of inflammatory infiltrates constitute early events in the disease course and are considered specific pathogenic features of MS (239). Sequential MRI analyses revealed that subtle changes in white matter areas can be detected prior to apparent lesions (240). Reduction of macromolecular material and a focal increase of free water could be detected as early as several months before the active lesion occurred. The study’s authors concluded that the reduction of magnetization exchange rate was likely to result from primary myelin injury (241). Furthermore, genome-wide epigenetic differences in the DNA methylation status have been reported between normal appearing white matter derived from MS patients and non-diseased control brains (242). The observed hypermethylation and subsequent diminished expression of loci in MS affected brains included genes that control oligodendrocyte survival (*BCL2L2* and *NDRG1*), suggesting that augmented susceptibility to injury precedes inflammatory infiltration. Importantly, a recent study showed that oligodendrocyte death is sufficient to induce encephalitogenic T cell responses and subsequent neuroinflammation *in vivo* (243). It is therefore conceivable that primary death of oligodendrocytes of yet unknown cause leads to subsequent uptake of myelin-derived antigenic debris by CNS-resident APCs and the following mounting of a myelin-specific adaptive response (244). The non-canonical autophagy pathway LAP may facilitate uptake and ensuing presentation of myelin protein-derived antigenic constituents, thus promoting local reactivation of encephalitogenic T_H_ cells.

Particularly early MS lesions depict enhanced gene expression related to ROS production. The most pronounced changes were found for transcripts encoding subunits of the NADPH oxidase complex 2 (NOX2), i.e., CYBA, CYBB, and NCF1, supporting the concept of oxidative stress as a pivotal pathogenic factor particularly early during the disease. NOX2 complex expression is detectable in up to 20% of myeloid cells, predominantly localized in areas of initial tissue damage at the edge of actively demyelinating lesions and is associated with the presence of CNS-infiltrating T cells suggesting that NOX2 expression by myeloid cells may contribute to CNS inflammation *via* recruitment or expansion of T cells within MS lesions (245). NOX2 is expressed in macrophages, DCs, microglia, neutrophils, and eosinophils (246). In resting conditions, the enzyme complex is highly glycosylated and resides in intracellular secondary granules and, at lower amounts, in the plasma membrane. After a triggering event, its subunits gp91 and p22 are translocated to the plasma membrane at the site of the forming phagosome, and the cytosolic subunits (p40, p47, p67) are recruited to assemble the active NOX2 complex (247). NOX2 function, generation of ROS, has initially been linked to killing of intraphagosomal pathogens by providing the adequate environment for protease function. In professional APCs, whose principal function is to take up, process and present antigens in order to initiate and shape adaptive immune responses rather than pathogen killing or clearance of cell debris, NOX2 expression was shown to regulate antigen presentation due to its ability to control the level of antigen degradation by altering phagosomal pH levels (248). Indeed, DCs lacking NOX2 show enhanced phagosomal acidification and increased antigen degradation, resulting in impaired cross presentation (248). In addition to cell type-dependent tuning of phagosomal acidification, NOX2 has recently also been implicated in regulating serine and cysteine proteases (cathepsins B, L, and S) within phagosomes (249) and it has been suggested that increased hydrolysis of critical regions within the encephalitogenic MOG_35–55_ antigen by cysteine cathepsins in the early phagosome contribute to the reduced incidence and delayed onset of EAE reported in NOX2-deficient mice (250). It is conceivable that NOX2 as essential component of the non-canonical autophagy pathway LAP not only affects levels of phagosomal proteolysis as previously shown, but facilitates the induction of an alternative pathway for antigen loading onto MHC class II molecules, thereby contributing to CD4^+^ T cell-mediated augmentation of autoimmune CNS inflammation.

Altogether, the aforementioned studies suggest that the autophagic machinery could potentially contribute to the initiation and maintenance of CD4^+^ T cell-driven CNS tissue injury through delivery of CD4^+^ T cell antigens into MIICs.

## Outlook: Autophagy Pathways in CNS Autoimmunity Beyond Antigen Presentation and Lymphocyte Survival

There is a profound body of evidence showing that mitochondrial dysfunction is a commonly observed feature in active MS plaques (194, 251–253). In lesions of patients suffering from acute MS, immunohistochemical analyses revealed mitochondrial defects selectively in respiratory chain complex IV subunit COX-I. Mitochondrial damage was observed in oligodendrocytes and axons, but was not present in macrophages or microglia (251). Mitochondria, while being a primary source thereof, are at the same time particularly susceptible to free radical-mediated damage and respiratory chain complex IV subunit COX-I expression is in part regulated by posttranslational modification of mRNA by nitric oxide (NO) (254–256). It has been suggested that myeloid cell-derived secretion of active mediators including NO in close proximity to oligodendrocytes mediates selective COX-I degradation with subsequent mitochondria impairment (221, 257–260). NMDA-receptor signaling has been shown to induce NADPH oxidase (NOX) activity in oligodendrocyte precursor cells and the subsequent production of ROS can drive both further oligodendrocyte differentiation and myelination (261, 262).

Malregulated oligodendrocyte-intrinsic events may also contribute to ROS-mediated mitochondrial damage in oligodendrocytes during MS. Mitochondrial homeostasis (mitostasis) and quality control is ensured by consecutive cycles of fusion and fission tightly coupled to mitophagy-mediated removal of aberrant organelles (263, 264) and, complex IV deficiency as well as concomitant cellular energy deficit can in part be compensated for by mitochondrial hyperfusion (265). It is therefore conceivable that malfunctioning of the autophagic axis within this regulatory network leads to disturbance of compensatory mechanisms during free radical induced COX-I deficiency (194, 263). Interestingly, as opposed to the observed functional and morphological impairment of mitochondria in highly active lesions, older and inactive lesions depict enhanced mitochondrial count and activity (266, 267).

In comparison to other cell types, neurons depict an unique geometry and organelles such as mitochondria are also needed in distal axonal regions afar from the neuronal soma where mitophagy-mediated lysosomal degradation takes place (268, 269). Duly distribution of mitochondria within axons is critical for neuronal physiology and the constant turnover of dysfunctional specimens in these post-mitotic cells requires well-regulated motor-protein-driven trafficking and anchoring of mitochondria alongside microtubules (269). Temporary increase in mitochondria number, size, and activity as well as subcellular re-location is believed to constitute an axonoprotective mechanism in the light of an elevated energy consumption during chronic axonal injury (270). This compensatory mitochondrial response relies on anchoring molecule syntaphilin, which mediates docking of mitochondria through interaction with microtubules since knockout of syntaphilin results in exacerbated degeneration of demyelinated neurons (270). Syntaphilin-anchored mitochondria have been reported to also recruit Parkin for subsequent mitophagy (268).

In addition to COX-I deficiency observed in acute MS lesions of the white matter, progressive disease also features mitochondrial alterations in deeper cortical areas reminiscent of prototypic neurodegenerative disorders with autophagy-associated pathoetiology such as Alzheimer’s disease (AD) and Parkinson’s disease (271, 272). Patients suffering from SPMS show significantly higher levels of clonally expanded mtDNA deletions in neurons of layer VI as compared to age-matched controls (272). Increased clonally expanded mtDNA deletions were also observed in layer VI adjacent subcortical white matter which suggests that in both instances soluble factors such as inflammation-associated free radicals in active lesions mediate mitochondrial degeneration. Although this needs to be clearly addressed experimentally, the possibility has been raised that accumulations of mtDNA deletions during MS might also yield mutations that could compromise autophagy-mediated quality control of mitochondria (194).

In the process of ATP generation by mitochondrial oxidative phosphorylation, ROS arise that in order to maintain cellular integrity and to prevent oxidative stress, need to be eliminated by means of several antioxidants such as NADPH, cytochrome *c*, and most importantly glutathione (273). However, aside from their detrimental role when produced in large quantities, if not exceedingly present, ROS, occupy also important signaling functions within the cell, interact with redox-sensitive proteins, and work toward maintaining cellular homeostasis in response to cell stress (274). Also Atgs have been described to be sensitive to ROS-mediated posttranslational modification. For example, Atg4 is oxidized and thereby inactivated by H_2_O_2_, which subsequently facilitates conjugation of LC3 to membranes (275). Furthermore, redox-sensitive nuclear transcription factor and histone deacetylase sirtuin 1 (SIRT1), which is highly expressed in neurons, promotes autophagic activity directly by complexing and deacetylation of essential autophagy-machinery proteins and indirectly by deacetylation of FoxO members which in turn leads to promotion of autophagy (274, 276).

Portending oxidative damage, strong immunoreactivity for oxidized phospholipids in neurons of active cortical MS lesions has been described to be specific to MS and was not observed to the same degree in tuberculous meningitis, AD, and age-matched controls (239). In accordance with these observations, antioxidant enzymes such as superoxide dismutase 1 and 2, heme oxigenase 1, and catalase have been shown to be increased in active MS lesions. Interestingly, this upregulation of antioxidant defense molecules was particularly present in astrocytes and myelin-containing macrophages but not in oligodendrocytes (277). It has been suggested before that the extreme susceptibility of oligodendrocytes to ROS-mediated damage is in part due to insufficient compensatory expression of protective enzymes (277, 278).

Increased mitochondrial-derived ROS accumulation and concomitant oxidative damage is believed to constitute a key feature of cellular aging and this process appears to be accelerated and augmented during MS particularly in oligodendrocytes and neurons (239, 279, 280). In line with this, treatment of aged mice with mTORC1 inhibitor and MA inducer rapamycin increased longevity in mice (281). It is likely that at least in part this effect was mediated by the compound’s capability to bridle injurious mitochondrial ROS production and mtDNA accumulation per induction of mitophagy (282).

Whole organelles such as mitochondria are not targeted for lysosomal degradation *via* LAMP-2A-dependent CMA. However, during cell stress, CMA does participate indirectly in mitochondrial quality control in neurons by removing non-functional PARK7, a redox-sensitive glycase with antioxidative properties (283).

In order to target autophagy pathways for therapeutic purposes in MS it will be crucial to fully elucidate the degree to which individual pathways (e.g., MI, MA, CMA, and LAP) contribute to pathology. This will be challenging since impairment of one pathway may lead to both, compensatory responses and concomitant attenuation in the remaining pathways (145). To this end, robust methods need to be established in order reliably distinguish and monitor discrete autophagy pathways. Taken together, these data suggest that regenerative and protective processes in oligodendrocytes and demyelinated axons are highly reliant on meticulously functioning mitochondrial quality control, during which autophagic pathways play a pivotal role (194, 269). Future research will show whether the autophagic machinery, in addition to its function in regulating innate and adaptive immune responses, interferes with neurobiological features of MS such as axonal degeneration.

## Author Contributions

Both authors have made substantial, intellectual, and equally valuable contribution to the work and approved it for publication.

## Conflict of Interest Statement

The authors declare that the research was conducted in the absence of any commercial or financial relationships that could be construed as a potential conflict of interest.
